# Effect of Domperidone on Insufficient Lactation in Puerperal Women: A Systematic Review and Meta-Analysis of Randomized Controlled Trials

**DOI:** 10.1155/2012/642893

**Published:** 2012-02-07

**Authors:** Alla Osadchy, Myla E. Moretti, Gideon Koren

**Affiliations:** Motherisk Program, Division of Clinical Pharmacology and Toxicology, Hospital for Sick Children, Toronto, ON, Canada M5G 1X8

## Abstract

*Background*. There is a controversy within the medical community regarding the role of domperidone as a galactagogue and the drug has been removed from the US market owing to safety concerns. *Objective*. To perform a systematic review and meta-analysis of the available data assessing the effect of domperidone on breast milk production in women experiencing insufficient lactation. *Study Selection*. Randomized controlled trials (RCTs) examining the effect of domperidone on breast milk production of puerperal women were eligible for inclusion. *Data Analysis*. Absolute and relative changes from baseline were calculated for individual studies and pooled using a random effects model. *Results*. Three RCTs including 78 participants met the inclusion criteria. All showed a statistically significant increase in breast milk production following treatment with domperidone. The analysis of pooled data demonstrated a statistically significant relative increase of 74.72% (95%  CI = 54.57; 94.86, *P* < 0.00001) in daily milk production with domperidone treatment compared to placebo. No maternal or neonatal adverse events were observed in any of the trials. *Conclusions*. Evidence from a few small RCTs of moderate to high quality suggests that domperidone produces a greater increase in breast milk supply than placebo.

## 1. Introduction

The benefits of breastfeeding are well recognized for both the mother and baby; thus, efforts should be made to promote initiation, duration, and exclusivity of breastfeeding [1]. The recently published survey of Canadian women who gave birth and were residing with their infants at the time of the interview has found that breastfeeding intention and initiating rates were fairly high, 90% and 90.3%, respectively, among women of this representative sample [2]. However, reported exclusive breastfeeding rates at three and six months fell substantially—51.7% and 14.4%. While factors that affect breastfeeding success are multiple and nonmodifiable at times, the early recognition and timely management of modifiable risk factors is warranted to improve lactation performance [3]. Various nonpharmacological interventions have been shown to be effective and hence are incorporated in the current clinical recommendations for promoting breastfeeding [1]. Among them are individual and group breastfeeding education provided by lactation specialists, peer counseling, in-person, or telephone support. Pharmacological interventions to improve lactation, mainly dopamine antagonists, are usually recommended only after nonpharmacological modalities have failed, and this is largely due to scarcity of available evidence and potential safety issues with pharmaceutical galactagogues [4, 5].

Domperidone, a peripheral dopamine receptor antagonist, is believed to enhance breast milk production by increasing prolactin secretion [6–8]. It has a favorable safety profile when compared to metoclopramide, another dopamine receptor antagonist, with only rare extra-pyramidal side effects owing likely to poor blood-brain barrier penetration of domperidone [9–11]. The drug is well tolerated with relatively few side effects reported including headache, dry mouth, and abdominal cramps [10, 12]. While domperidone is not available for any indication in the United States due to arrhythmia concerns, it is approved in Canada and other countries as a prokinetic agent. Moreover, there is a worldwide experience with domperidone in treating nausea and vomiting. The use of domperidone as a galactagogue, hence, represents an “off-label” indication.

In 2004, the United States Food and Drug Administration (FDA) issued an advisory against the use of domperidone as a milk enhancer due to safety concerns [13]. There have been a few reports of cardiac arrhythmia and sudden death in cancer patients treated with intravenous domperidone which are often cited in the literature [14]. Rapid intravenous administration or high doses of domperidone as well as concurrent hypokalemia might be significant contributors to these adverse outcomes leading to discontinuation of the intravenous route of administration. A single case report of reversible QT prolongation associated with oral domperidone administration has been published [15]. In neonates, oral administration of domperidone was associated with QT prolongation [16]. Whereas the potential pro-arrhythmic effect of domperidone should not be ignored, the FDA concern over the use of domperidone for promoting lactation has been regarded by lactation experts as a gross overestimation. Available pharmacokinetic data, although limited, indicates minimal excretion of domperidone into breast milk with extremely low (less than 0.01% of the maternal weight-adjusted dose) infant exposure via breast milk [6–8, 12]. No side effects have been reported in exposed infants. The American Academy of Pediatrics lists domperidone as compatible with breastfeeding [17].

Nevertheless, there is a controversy regarding the role of domperidone as a galactagogue: some authors claim no or little effectiveness, largely due to limitations of available data [18] while other researchers suggested that domperidone is a galactagogue of choice based on evidence available [4]. This situation might be a source of confusion in the medical community and, therefore, may compromise clinical management decisions.

 The objective of our study was to perform a systematic review and meta-analysis of the available data assessing the effect of domperidone on breast milk supply in women experiencing insufficient breast milk production.

## 2. Methods

### 2.1. Eligibility Criteria

Randomized controlled trials (RCTs) examining the effect of domperidone on breast milk production were considered for inclusion. We utilized the PICO format (population, intervention, comparison, and outcome) to develop our clinical question, guide the literature search, and assess eligibility of potentially relevant studies. The population of interest was puerperal women who had experienced insufficient lactation after delivery. We accepted any definition of insufficient lactation, with the most common definition being milk supply below the infant's daily oral feeding requirements. The intervention considered for this paper was domperidone treatment to augment lactation; the comparator considered was placebo or no treatment. The outcome of interest was percent change in daily breast milk volume after domperidone treatment.

### 2.2. Search Strategy

The following electronic databases were searched: Ovid MEDLINE(R) (1948 to May 2011), EMBASE (1947 to May 2011), and Cochrane Library, with no restrictions on language or year of publication. The last search was run on May 31 2011. Our search strategy included the following National Library of Medicine Medical Subject Headings (MeSH) terms: “domperidone” combined with “lactation” OR “milk production” OR “galactagogue” OR “breastfeeding.” The search was limited further to human data and clinical trials. Reference lists of relevant review papers and all selected articles were hand searched to identify additional trials.

### 2.3. Study Selection and Quality Assessment

Literature search and eligibility assessment was performed independently by two reviewers. One reviewer extracted the data and performed quality assessment of included trials. The second reviewer checked the extracted data and quality assessment. Disagreements in judgment between reviewers were resolved by discussion. The following data was extracted: characteristics of trial participants (number, inclusion criteria), type of intervention (dose and duration of domperidone or placebo treatment), outcome measure (type and assessment tool), and maternal and neonatal adverse effects reported.

Study quality was assessed using the GRADE (grading of recommendations, assessment, development, and evaluation) system [21]. The GRADE system was developed by a widely representative group of scientists and adopted by the Cochrane Collaboration to assess the quality of evidence for outcomes reported in systematic reviews. Each individual study was rated as that of high, moderate, low, or very low quality. The Cochrane Collaboration's tool has been applied to assess risk of bias across studies. The following domains were evaluated—sequence generation, blinding, allocation concealment, incomplete outcome data, selective outcome reporting, and other sources of bias. Randomized controlled trials are generally rated as a high quality but might be downgraded. Factors that may decrease the quality of evidence include serious limitations in design, imprecision of results, unexplained heterogeneity, and indirectness of evidence and high probability of publication bias.

### 2.4. Statistical Analysis

The primary effect measure for this paper was the change in daily breast milk volume from baseline to the end of the treatment presented as a mean difference and standard deviation. Absolute and relative changes from baseline were recorded for individual studies. Absolute mean differences in daily breast milk volumes before and after treatment were extracted from individual studies. Relative mean differences were calculated as percentage change from baseline.

When the standard deviations of the absolute changes from baseline were not available from individual studies, we imputed them as described in detail in the Cochrane Handbook [22–24]. In brief, we calculated correlation coefficients from one available study which reported the means and standard deviations for change in breast milk volume from baseline [8]. Using the imputed correlation coefficients values, we thereafter calculated a change from baseline standard deviations for the other studies with missing standard deviations [19, 20]. A sensitivity analysis was performed utilizing the lowest and highest values of the correlation coefficient to determine the robustness of the results.

The standard deviations of relative change (%) were calculated as SD_relative  change_ = SD_absolute  change_/breast  milk  volume_baseline_. Pooled estimates of the weighted mean differences and 95% CI were calculated using a random effects model. The *I* ^²^ statistic was used to assess the extent of heterogeneity among studies. A priori subgroup analyses were not planned. Due to insufficient number of studies, a formal assessment of reporting bias by visual inspection of a funnel plot was not possible.

## 3. Results

### 3.1. Study Selection

**Figure 1:**
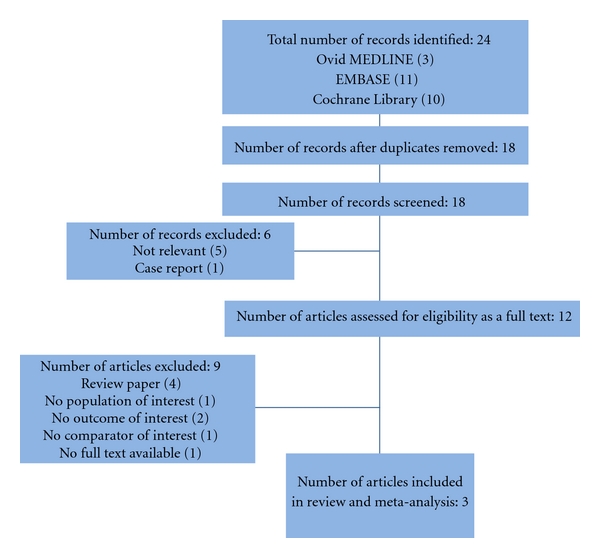
Flow chart of selected studies.

The literature search retrieved a total of 24 citations (Figure 1). After duplicate publications were eliminated, 18 remaining abstracts were screened for eligibility. Of these, six were excluded (five were deemed not relevant and one was a case report). The full text of the remaining 12 citations was analyzed further in detail. Nine papers were excluded due to various reasons. Three studies met the inclusion criteria and were included in the systematic review and meta-analysis [8, 19, 20].

### 3.2. Study Characteristics

All three studies selected for this review were randomized, placebo-controlled trials with a total of 78 patients enrolled (37 in domperidone group and 41 in placebo group) [8, 19, 20].  Table 1 summarizes characteristics of included studies. All participants have experienced inadequate breast milk production postpartum and, therefore, were randomized to domperidone or placebo. Of note, all mothers were enrolled after a few weeks postpartum allowing time to establish lactation and/or receive appropriate lactation support. However, only one study mentioned extensive lactation counseling prior to enrolment [8].

The dose of domperidone used across the studies was 30 mg/d (10 mg orally 3 times daily). The length of the treatment ranged from 7 to 14 days. All studies reported the change in daily milk production from baseline to the end of the treatment. In Petraglia et al. [19], the mothers breastfed their full-term infants, and thus daily milk volumes were assessed by weighing the babies before and after breastfeeding. In two other studies [8, 20], the mothers pumped breast milk to feed their preterm babies and the amount of milk expressed was recorded.

### 3.3. Methodological Quality of Included Studies

Table 2 displays the summary of risk of bias for individual studies included in the meta-analysis. Two of the studies, by Da Silva et al. [8] and Campbell-Yeo et al. [20], were ranked as having low risk of bias. The description of randomization, allocation concealment, blinding, and reporting in these two papers was judged as adequate. Da Silva et al. [8], however, reported incomplete or nonreturned records for three out of 11 participants in the domperidone group which represents missing data for >25% of participants. Overall, both studies were judged as free of serious limitations and were graded as high-quality evidence.

The study done by Petraglia et al. [19], to the contrary, did not provide sufficient information on sequence generation and allocation concealment. Furthermore, the study is described as a double-blind trial; however, there is no information whether placebo and active drug were of similar appearance and taste. It is also unclear from the paper whether all women randomized initially completed the trial. Given the above-mentioned limitations in the study design and implementation, Petraglia et al. was downgraded from high- to moderate-quality evidence. 

### 3.4. Results of Individual Studies

Three included RCTs evaluated the effect of domperidone on a daily breast milk volume in the women with insufficient lactation in comparison to placebo.

Da Silva et al. reported that after 7-day treatment, the mean daily milk volume had increased by 49.5 (SD = 29.4) mL/day in the domperidone group compared to 8.0 (SD = 39.5) mL/day in the placebo group [8]. Similarly, Petraglia et al. demonstrated that, following 10-day treatment, daily milk yield was significantly higher in a small group of domperidone-treated mothers than that of the placebo-treated group [19]. The mean increase in daily milk yield was 326 (imputed SD = 21.4) mL/day after domperidone versus 63 (imputed SD = 23.7) mL/day after placebo treatment.

Finally, a significant increase in daily breast milk production was found in Campbell-Yeo et al. [20]: mean increase of 195.8 (imputed SD = 98.1) mL/day after a 14-day course of domperidone compared to 33.1 (imputed SD = 83.2) mL/day in a placebo-treated group [20].

Overall, in absolute values, all three studies had shown a statistically significant increase from baseline in breast milk production following treatment with domperidone.

**Figure 2:**
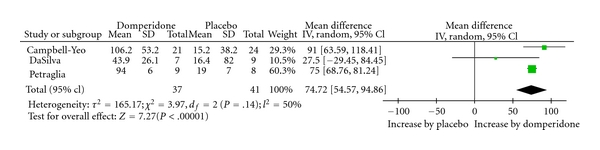
Percent change in milk volume with domperidone treatment.

Due to substantial differences in baseline milk volumes across the studies, the relative changes from baseline were calculated and used to estimate the pooled effect of domperidone (Figure 2).

### 3.5. Pooled Analysis

The analysis of pooled data demonstrated a statistically significant increase, of 74.7% (95% CI = 54.6; 94.9, *P* < 0.00001) in daily milk production following treatment with domperidone while comparing to placebo. We observed a moderate statistical heterogeneity among the studies (*I* ^2^ = 50%).

## 4. Discussion

Our findings indicate that domperidone increases inadequate breast milk production in nursing mothers more effectively than placebo. A statistically significant increase in the mean change in daily breast milk volume from baseline was observed in all three studies comparing domperidone and placebo. This consistency of the domperidone effect across the studies enhances the confidence of its beneficial effect as a galactagogue. Importantly, no maternal or neonatal adverse events were observed in any of the three trials. Although not included in this analysis, the study by Wan and colleagues demonstrated a dose-response increase in milk production, further supporting our findings [12].

Another strength of the current meta-analysis lies in the fact that, despite the paucity of published reports on effectiveness of domperidone to promote lactation, we attempted to identify and include only randomized placebo-controlled studies which are regarded as higher quality evidence. Two out of the three included trials fulfilled the GRADE criteria for high-quality evidence [8, 20], while the third study [19] was downgraded to moderate-quality evidence due to insufficient details on study design and execution and, therefore, as having a potential risk of bias.

Based on the moderate-high quality of evidence from three RCTs, the pooled effect of a 75% increase from baseline in daily milk production following treatment with domperidone is deemed to be clinically meaningful.

Our study has several limitations. Only three eligible studies were found with small sample sizes (17, 16, and 45 participants in each trial, resp.). It has been suggested that small trials with an insufficient number of participants and events may produce spurious treatment effects due to random error [25]. Furthermore, there have been reports showing that some meta-analyses become inconclusive when adjusted for random error risk [26, 27]. Hence, the calculation of optimal information size (similar to the concept of sample size calculation for individual studies) and the use of appropriate statistical tools (i.e., trial sequential analysis) have been advocated to judge results of meta-analysis as reliable and conclusive. 

On the other hand, it is unclear how many studies are needed to be included in meta-analysis to render results trustworthy. Moreover, some researchers have demonstrated that meta-analysis with a fewer trials do produce robust results consistent with long-run findings [28]. However, it is difficult to foresee which results might be changed by subsequent large-scale trials. Nonetheless, owing to the above-mentioned limitations, our findings must be interpreted with caution, and generalizable recommendations might be still premature.

Additionally, a moderate statistical heterogeneity was found to exist across the studies' results. While all three trials have utilized the same doses of domperidone (or placebo) and reasonably similar duration of treatment, the differences in study populations (mothers of preterm versus full-term infants, breastfeeding or pumping their milk) and outcome measurement instruments (increase in milk supply versus infant weight gain) are likely to explain the observed heterogeneity. We have used a random effects model for the pooled estimate to deal with statistical heterogeneity. However, too few studies available precluded subgroup analyses to further explore the observed heterogeneity. We believe, though, that the selected trials were methodologically sound to combine in the present meta-analysis. The clinical relevance of this modest heterogeneity is probably not meaningful as there is a considerable consistency in domperidone effect across individual studies and no biological reason to suspect that the opposite direction effect might be true. Still, it is sensible to investigate potential sources of heterogeneity as more research on this topic becomes available.

One methodological challenge we encountered in the present meta-analysis is not uncommon and thus deserves special mention. The issue is related to handling missing variance estimates data in primary studies included in meta-analysis. Two out of three RCTs selected for our review failed to provide standard deviations (SDs) for changes from baseline which we selected as a primary effect measure. There have been several methods proposed to impute missing variance estimates for continuous outcomes [22–24]. Since Da Silva et al. [8] reported SDs, we were able to calculate the correlation coefficient, a measurement of similarity between the baseline and final measurements across participants from this study, and then apply the calculated value to impute a change-from-baseline standard deviation for two other studies included in our meta-analysis. In general, a correlation coefficient of zero indicates no correlation which is unusual for clinical outcomes as we expect certain degree of association between measurements within an individual. Similarly, a correlation coefficient of one is unlikely due to certain variability present within an individual. The calculated correlations obtained from Da Silva et al. were reasonably similar and close to 1 for the domperidone and placebo groups (0.97 and 0.78, resp.). We used an average correlation coefficient of 0.875 to impute the missing change-from-baseline standard deviations for the remaining studies. A sensitivity analysis was performed utilizing the lowest and highest values of the correlation coefficient and repeating the analysis. This did not change our overall conclusion as the pooled estimates and confidence intervals were not significantly changed in terms of magnitude or directionality (data not shown). Although a certain degree of uncertainty exists regarding the accuracy of the results derived from this approach, there is a growing body of the literature indicating the validity of results from meta-analyses utilizing various imputation methods [29, 30].

## 5. Conclusions 

Currently available data from a few small randomized controlled trials suggest that domperidone produces greater increase in breast milk supply than that found with placebo in some puerperal women with insufficient milk production. These results, however, should be interpreted in the context of the limitations of available data. Additional randomized clinical trials of adequate sample size are desirable and might have an impact on our confidence in the estimate of domperidone effect as a galactagogue. In the realm of clinical practice, however, while the balance between desirable and undesirable effects often guides treatment decisions, the current analysis supports consideration that domperidone might be an effective treatment option for selected women with inadequate lactation. It appears to be prudent though to try nonpharmacological interventions, for example, maternal lactation education, first [1, 5].

## Figures and Tables

**Table 1:** Characteristics of trials included in analysis.

Reference	N of participant, Intervention/placebo groups	Inclusion criteria	Domperidone dose	Domperidone duration of treatment	Outcome	Outcome assessment
Petraglia et al. [19] (1985)	9/8	Premiparous mothers of term infants with insufficient lactation^a^ 2 weeks post partum	30 mg/day	10 days	Daily breast milk yield, before and after treatment, mL/day	By weighing the infants before and after breastfeeding using an electronic integrating scale and summarizing the single milk yields for the day

Da Silva et al. [8] (2001)	7/9	Mothers of preterm infants with low milk production^b^	30 mg/day	7 days	Daily breast milk volume, before and after treatment, mL/day	Mechanically expressed breast milk by using a double collecting pump

Campbell-Yeo et al. (2010) [20]	21/24	Mothers of preterm infants (<31 weeks gestation) with lactation failure^c^ ≥3 wks after delivery	30 mg/day	14 days	Daily breast milk volume, before and after treatment, mL/day	Mechanically expressed breast milk by using a double collecting system

^a^insufficient lactation defined as milk yields at least 30% lower than those reported as normal

^b^low milk production defined as not meeting the infant's daily oral feeding requirements

^c^lactation failure defined as not of the following: a decreasing milk supply by >30% from peak volume based on maternal count or inability to meet the daily nutritional intake of the infant.

**Table 2:** Methodological quality of RCTs included in the meta-analysis.

Studies, year	Sequence generation	Allocation concealment	Blinding of participants and personnel	Blinding of outcome assessment	Incomplete outcome data	Selective outcome reporting	Other bias
Petraglia et al. [19] (1985)	Insufficient information/unclear risk of bias	Insufficient information/unclear risk of bias	Insufficient information/unclear risk of bias	Insufficient information/unclear risk of bias	Insufficient information/unclear risk of bias	Insufficient information/unclear risk of bias	No/low risk of bias

Da Silva et al. [8] (2001)	Yes/low risk of bias	Yes/low risk of bias	Yes/low risk of bias	Yes/low risk of bias	Insufficient information/unclear risk of bias	No/low risk of bias	No/low risk of bias

Campbell-Yeo et al. [20] (2011)	Yes/low risk of bias	Yes/low risk of bias	Yes/low risk of bias	Yes/low risk of bias	No/low risk of bias	No/low risk of bias	No/low risk of bias
